# Crystal structure of (*E*)-3-allyl-2-sulfanyl­idene-5-[(thio­phen-2-yl)methyl­idene]thia­zolidin-4-one

**DOI:** 10.1107/S2056989015010166

**Published:** 2015-05-30

**Authors:** Rahhal El Ajlaoui, El Mostapha Rakib, Souad Mojahidi, Mohamed Saadi, Lahcen El Ammari

**Affiliations:** a Laboratoire de Chimie Organique et Analytique, Université Sultan Moulay Slimane, Faculté des Sciences et Techniques, Béni-Mellal, BP 523, Morocco; b Laboratoire de Chimie du Solide Appliquée, Faculté des Sciences, Université Mohammed V, Avenue Ibn Battouta, BP. 1014, Rabat, Morocco

**Keywords:** crystal structure, rhodanine derivative, 2-thioxa­thia­zolidin-4-one, hydrogen bonding, π–π inter­actions

## Abstract

Mol­ecules of the title compound, C_11_H_9_NOS_3_, are built up by one thio­phene and one 2-thioxa­thia­zolidin-4-one ring which are connected by a methyl­ene bridge. In addition, there is an allyl substituent attached to nitro­gen. The two rings are almost coplanar, making a dihedral angle between them of 0.76 (11)°. The allyl group is oriented perpendicular to the mean plane through both ring systems. The crystal structure exhibits inversion dimers in which mol­ecules are linked by pairs of C—H⋯O hydrogen bonds. Additional π–π inter­actions between neighboring thio­phene and 2-thioxa­thia­zolidin-4-one rings [inter­centroid distance = 3.694 (2) Å] lead to the formation of a three-dimensional network.

## Related literature   

For pharmacological activities such as anti­microbial and anti-inflammatory of aryl­idene derivatives of rhodanine (2-thioxo-1,3-thia­zolidin-4-one), see: Soltero-Higgin *et al.* (2004 ▸); Hu *et al.* (2004 ▸); Nasr & Said (2003 ▸); Johnson *et al.* (2001 ▸); Sortino *et al.* (2007 ▸); Insuasty *et al.* (2010 ▸); Tomasic & Masic (2009 ▸).
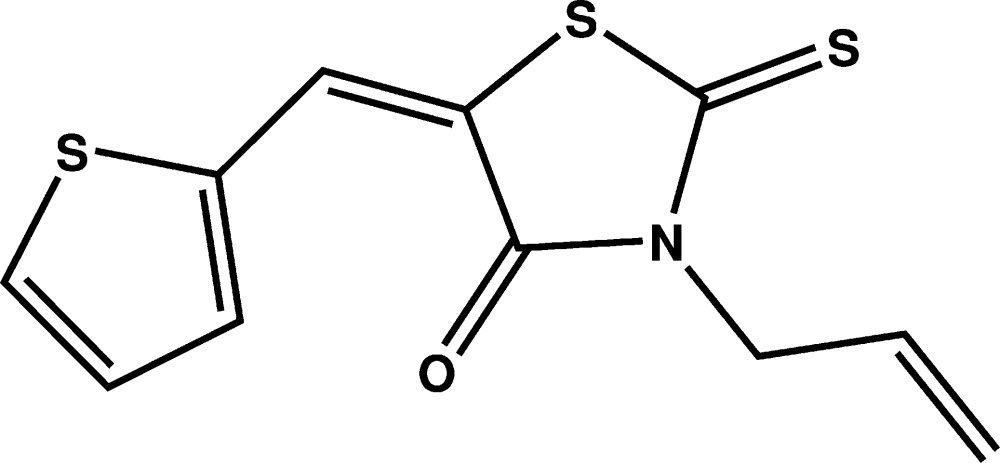


## Experimental   

### Crystal data  

C_11_H_9_NOS_3_
*M* *_r_* = 267.37
Triclinic, 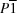

*a* = 6.7342 (2) Å
*b* = 7.3762 (2) Å
*c* = 13.2917 (5) Å
α = 79.386 (2)°
β = 80.104 (2)°
γ = 68.908 (1)°
*V* = 601.44 (3) Å^3^
*Z* = 2
Mo *K*α radiation
μ = 0.59 mm^−1^
*T* = 296 K
0.37 × 0.35 × 0.28 mm

### Data collection  

Bruker X8 APEX diffractometer
Absorption correction: multi-scan (*SADABS*; Bruker, 2009 ▸) *T*_min_ = 0.700, *T*_max_ = 0.746
25223 measured reflections
3514 independent reflections
2557 reflections with *I* > 2σ(*I*)
*R*_int_ = 0.042

### Refinement  

*R*[*F* ^2^ > 2σ(*F* ^2^)] = 0.043
*wR*(*F* ^2^) = 0.111
*S* = 1.07
3514 reflections
145 parameters
H-atom parameters constrained
Δρ_max_ = 0.35 e Å^−3^
Δρ_min_ = −0.26 e Å^−3^

### 

Data collection: *APEX2* (Bruker, 2009 ▸); cell refinement: *SAINT* (Bruker, 2009 ▸); data reduction: *SAINT*; program(s) used to solve structure: *SHELXS97* (Sheldrick, 2008 ▸); program(s) used to refine structure: *SHELXL97* (Sheldrick, 2008 ▸); molecular graphics: *ORTEPIII* (Burnett & Johnson, 1996 ▸) and *ORTEP-3 for Windows* (Farrugia, 2012 ▸); software used to prepare material for publication: *PLATON* (Spek, 2009 ▸) and *publCIF* (Westrip, 2010 ▸).

## Supplementary Material

Crystal structure: contains datablock(s) I. DOI: 10.1107/S2056989015010166/im2466sup1.cif

Structure factors: contains datablock(s) I. DOI: 10.1107/S2056989015010166/im2466Isup2.hkl

Supporting information file. DOI: 10.1107/S2056989015010166/im2466Isup3.cml

**Fig. 1.**
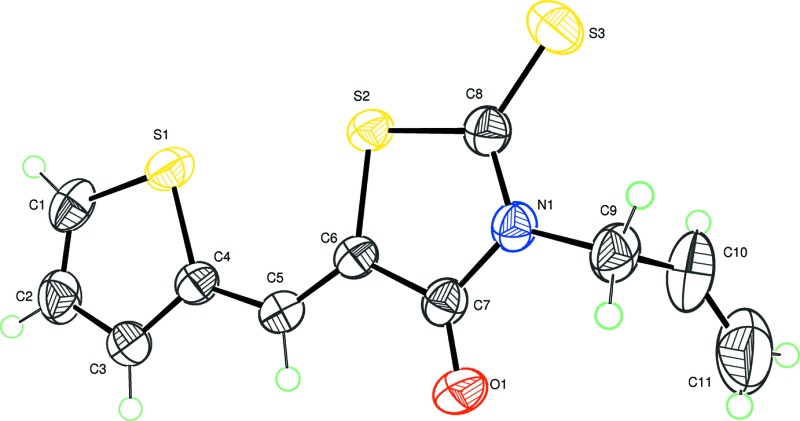
Molecular structure of the title compound with displacement ellipsoids drawn at the 50% probability level. H atoms are represented as small circles of arbitrary radius.

. DOI: 10.1107/S2056989015010166/im2466fig1.tif
Mol­ecular structure of the title compound with displacement ellipsoids drawn at the 50% probability level. H atoms are represented as small circles of arbitrary radius.

**Fig. 2.**
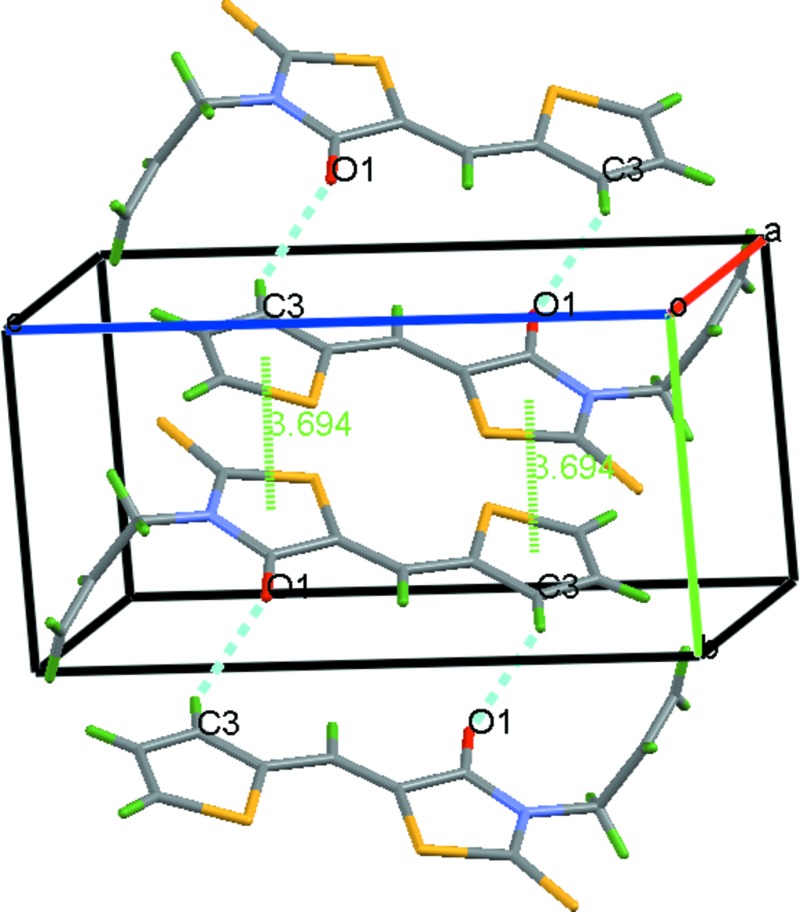
Partial crystal packing of the title compound showing hydrogen bonds and π–π interactions between molecules.

. DOI: 10.1107/S2056989015010166/im2466fig2.tif
Partial crystal packing of the title compound showing hydrogen bonds and π–π inter­actions between mol­ecules.

CCDC reference: 1403058

Additional supporting information:  crystallographic information; 3D view; checkCIF report

## Figures and Tables

**Table 1:** Hydrogen-bond geometry (, )

*D*H*A*	*D*H	H*A*	*D* *A*	*D*H*A*
C3H3O1^i^	0.93	2.54	3.304(3)	140

Symmetry code: (i) 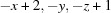.

## supplementary crystallographic information

### S1. Comment

Rhodanine (2-thioxo-1,3-thiazolidin-4-one) is the key structural feature of a very important group of heterocyclic compounds for drug discovery programs. Arylidene derivatives of rhodanine have attracted great interest for synthetic organic chemists due to the broad biological activities of this class of compounds including antimicrobial (Sortino *et al.*; 2007, Hu *et al.*, 2004), anti-inflammatory (Nasr & Said, 2003; Johnson *et al.* 2001), and antifungal (Sortino *et al.*, 2007; Insuasty *et al.*, 2010) properties. Additionally, rhodanine derivatives may potentially be used in the treatment of diabetes, obesity, Alzheimer's disease, cystic fibrosis, thrombocytopenia, cancer, sleep, mood and central nervous system disorders as well as chronic inflammation (Tomasic & Masic, 2009).

The two five-membered rings (C1–C4, S1 and C6– C8, N1, S2) forming the molecule are almost coplanar, with a maximum deviation of -0.023 (2) Å for C7 (Fig.1). The allyl group is oriented perpendicular to the mean plane through the thioxothiazolidine cycle as indicated by the torsion angle C10–C9–N1–C7 of 90.2 (3)°.

The cohesion of the crystal structure is ensured by C3–H3···O1 hydrogen bonds between molecules forming a dimers and π–π interactions between heterocycles [intercentroid distance = 3.69 (2) Å], forming a three-dimensional network as shown in Fig.2 and Table 1.

### S2. Experimental

To a solution of 3-allyl-2-thioxo-1,3-thiazolidin-4-one (1.15 mmol, 0.2 g) in 10 ml of THF methyl-2-(thiophen-2-ylmethylene)-5-oxopyrazolidin-2-ium-1-ide (1.38 mmol) was added. The mixture was refluxed for 8 h and was monitored by TLC. After the reaction was completed only one yellow spot (TLC R_f_ = 0.3, hexane/ethyl acetate 1:9) was generated cleanly. The solvent was evaporated *in vacuo*. The crude product was purified on silica using hexane: ethyl acetate (1/9) as eluent. The product was obtained as a yellow crystal solid (Yield: 55%, m.p.: 403 K).

### S3. Refinement

H atoms were located from the difference Fourier map and treated as riding with C–H = 0.97 Å and C–H = 0.93 Å for methylene and aromatic, respectively. All hydrogen atoms were included into the refinement with *U*_iso_(H) = 1.2 *U*_eq_ of the parent carbon atom.

### Crystal data

**Table:**

C_11_H_9_NOS_3_	*Z* = 2
*M**_r_* = 267.37	*F*(000) = 276
Triclinic, *P*1	*D*_x_ = 1.476 Mg m^−^^3^
*a* = 6.7342 (2) Å	Mo *K*α radiation, λ = 0.71073 Å
*b* = 7.3762 (2) Å	Cell parameters from 3514 reflections
*c* = 13.2917 (5) Å	θ = 3.0–30.0°
α = 79.386 (2)°	µ = 0.59 mm^−^^1^
β = 80.104 (2)°	*T* = 296 K
γ = 68.908 (1)°	Block, yellow
*V* = 601.44 (3) Å^3^	0.37 × 0.35 × 0.28 mm

### Data collection

**Table:**

Bruker X8 APEX diffractometer	3514 independent reflections
Radiation source: fine-focus sealed tube	2557 reflections with *I* > 2σ(*I*)
Graphite monochromator	*R*_int_ = 0.042
φ and ω scans	θ_max_ = 30.0°, θ_min_ = 3.0°
Absorption correction: multi-scan (*SADABS*; Bruker, 2009)	*h* = −9→9
*T*_min_ = 0.700, *T*_max_ = 0.746	*k* = −10→10
25223 measured reflections	*l* = −18→18

### Refinement

**Table:**

Refinement on *F*^2^	0 restraints
Least-squares matrix: full	Hydrogen site location: inferred from neighbouring sites
*R*[*F*^2^ > 2σ(*F*^2^)] = 0.043	H-atom parameters constrained
*wR*(*F*^2^) = 0.111	*w* = 1/[σ^2^(*F*_o_^2^) + (0.0384*P*)^2^ + 0.3716*P*] where *P* = (*F*_o_^2^ + 2*F*_c_^2^)/3
*S* = 1.07	(Δ/σ)_max_ < 0.001
3514 reflections	Δρ_max_ = 0.35 e Å^−^^3^
145 parameters	Δρ_min_ = −0.26 e Å^−^^3^

### Special details

**Table:**

Geometry. All e.s.d.'s (except the e.s.d. in the dihedral angle between two l.s. planes) are estimated using the full covariance matrix. The cell e.s.d.'s are taken into account individually in the estimation of e.s.d.'s in distances, angles and torsion angles; correlations between e.s.d.'s in cell parameters are only used when they are defined by crystal symmetry. An approximate (isotropic) treatment of cell e.s.d.'s is used for estimating e.s.d.'s involving l.s. planes.

### Fractional atomic coordinates and isotropic or equivalent isotropic displacement parameters (Å^2^)

**Table:**

	*x*	*y*	*z*	*U*_iso_*/*U*_eq_	
C1	0.1345 (4)	0.1860 (4)	0.70306 (19)	0.0518 (6)	
H1	−0.0046	0.2033	0.7342	0.062*	
C2	0.3105 (4)	0.0861 (4)	0.75114 (18)	0.0523 (6)	
H2	0.3058	0.0267	0.8192	0.063*	
C3	0.5018 (4)	0.0808 (3)	0.68783 (17)	0.0428 (5)	
H3	0.6372	0.0182	0.7094	0.051*	
C4	0.4669 (3)	0.1785 (3)	0.59025 (16)	0.0350 (4)	
C5	0.6327 (3)	0.1940 (3)	0.50925 (16)	0.0358 (4)	
H5	0.7718	0.1321	0.5264	0.043*	
C6	0.6167 (3)	0.2849 (3)	0.41203 (16)	0.0341 (4)	
C7	0.8086 (3)	0.2828 (3)	0.33909 (16)	0.0366 (4)	
C8	0.5339 (3)	0.4692 (3)	0.23605 (17)	0.0399 (5)	
C9	0.9117 (4)	0.4146 (4)	0.15934 (19)	0.0491 (6)	
H9A	0.8550	0.5408	0.1181	0.059*	
H9B	1.0355	0.4141	0.1876	0.059*	
C10	0.9801 (6)	0.2599 (5)	0.0927 (2)	0.0743 (9)	
H10	0.8740	0.2392	0.0646	0.089*	
C11	1.1737 (7)	0.1513 (6)	0.0701 (3)	0.1111 (16)	
H11A	1.2845	0.1674	0.0967	0.133*	
H11B	1.2038	0.0562	0.0272	0.133*	
N1	0.7483 (3)	0.3913 (3)	0.24437 (13)	0.0382 (4)	
O1	0.9933 (2)	0.2023 (3)	0.35541 (13)	0.0515 (4)	
S1	0.19548 (9)	0.27676 (9)	0.57905 (5)	0.04606 (16)	
S2	0.38596 (8)	0.41472 (8)	0.35129 (4)	0.03895 (14)	
S3	0.41977 (12)	0.59688 (12)	0.13403 (5)	0.0646 (2)	

### Atomic displacement parameters (Å^2^)

**Table:**

	*U*^11^	*U*^22^	*U*^33^	*U*^12^	*U*^13^	*U*^23^
C1	0.0437 (13)	0.0519 (14)	0.0552 (15)	−0.0171 (11)	0.0135 (11)	−0.0117 (11)
C2	0.0583 (15)	0.0566 (14)	0.0374 (12)	−0.0213 (12)	0.0061 (11)	−0.0020 (10)
C3	0.0425 (12)	0.0437 (12)	0.0389 (11)	−0.0123 (10)	−0.0042 (9)	−0.0028 (9)
C4	0.0343 (10)	0.0315 (10)	0.0392 (11)	−0.0108 (8)	−0.0029 (8)	−0.0065 (8)
C5	0.0316 (10)	0.0343 (10)	0.0406 (11)	−0.0101 (8)	−0.0032 (8)	−0.0054 (8)
C6	0.0304 (9)	0.0340 (10)	0.0377 (10)	−0.0111 (8)	−0.0022 (8)	−0.0060 (8)
C7	0.0337 (10)	0.0393 (11)	0.0389 (11)	−0.0156 (9)	−0.0002 (8)	−0.0077 (9)
C8	0.0402 (11)	0.0412 (11)	0.0394 (11)	−0.0151 (9)	−0.0030 (9)	−0.0063 (9)
C9	0.0447 (13)	0.0555 (14)	0.0469 (13)	−0.0242 (11)	0.0066 (10)	−0.0024 (10)
C10	0.084 (2)	0.086 (2)	0.0568 (17)	−0.0438 (19)	0.0266 (15)	−0.0216 (15)
C11	0.133 (4)	0.086 (3)	0.067 (2)	0.002 (2)	0.025 (2)	−0.0083 (19)
N1	0.0360 (9)	0.0424 (9)	0.0370 (9)	−0.0165 (8)	0.0006 (7)	−0.0051 (7)
O1	0.0298 (8)	0.0662 (11)	0.0532 (10)	−0.0130 (7)	−0.0035 (7)	−0.0032 (8)
S1	0.0340 (3)	0.0453 (3)	0.0515 (3)	−0.0083 (2)	−0.0008 (2)	−0.0031 (2)
S2	0.0291 (2)	0.0432 (3)	0.0410 (3)	−0.0101 (2)	−0.0029 (2)	−0.0024 (2)
S3	0.0557 (4)	0.0842 (5)	0.0448 (4)	−0.0184 (4)	−0.0133 (3)	0.0101 (3)

### Geometric parameters (Å, º)

**Table:**

C1—C2	1.347 (4)	C7—O1	1.208 (2)
C1—S1	1.701 (3)	C7—N1	1.400 (3)
C1—H1	0.9300	C8—N1	1.364 (3)
C2—C3	1.405 (3)	C8—S3	1.638 (2)
C2—H2	0.9300	C8—S2	1.743 (2)
C3—C4	1.377 (3)	C9—C10	1.468 (4)
C3—H3	0.9300	C9—N1	1.468 (3)
C4—C5	1.433 (3)	C9—H9A	0.9700
C4—S1	1.729 (2)	C9—H9B	0.9700
C5—C6	1.344 (3)	C10—C11	1.278 (5)
C5—H5	0.9300	C10—H10	0.9300
C6—C7	1.473 (3)	C11—H11A	0.9300
C6—S2	1.749 (2)	C11—H11B	0.9300
			
C2—C1—S1	112.36 (18)	N1—C8—S3	126.87 (17)
C2—C1—H1	123.8	N1—C8—S2	110.96 (16)
S1—C1—H1	123.8	S3—C8—S2	122.17 (13)
C1—C2—C3	113.0 (2)	C10—C9—N1	113.0 (2)
C1—C2—H2	123.5	C10—C9—H9A	109.0
C3—C2—H2	123.5	N1—C9—H9A	109.0
C4—C3—C2	112.6 (2)	C10—C9—H9B	109.0
C4—C3—H3	123.7	N1—C9—H9B	109.0
C2—C3—H3	123.7	H9A—C9—H9B	107.8
C3—C4—C5	124.61 (19)	C11—C10—C9	125.3 (4)
C3—C4—S1	110.43 (16)	C11—C10—H10	117.4
C5—C4—S1	124.96 (16)	C9—C10—H10	117.4
C6—C5—C4	129.47 (19)	C10—C11—H11A	120.0
C6—C5—H5	115.3	C10—C11—H11B	120.0
C4—C5—H5	115.3	H11A—C11—H11B	120.0
C5—C6—C7	121.35 (19)	C8—N1—C7	116.63 (17)
C5—C6—S2	128.78 (16)	C8—N1—C9	122.99 (19)
C7—C6—S2	109.86 (15)	C7—N1—C9	120.37 (18)
O1—C7—N1	123.06 (19)	C1—S1—C4	91.64 (11)
O1—C7—C6	126.9 (2)	C8—S2—C6	92.49 (10)
N1—C7—C6	110.02 (17)		

### Hydrogen-bond geometry (Å, º)

**Table:**

*D*—H···*A*	*D*—H	H···*A*	*D*···*A*	*D*—H···*A*
C3—H3···O1^i^	0.93	2.54	3.304 (3)	140

Symmetry code: (i) −*x*+2, −*y*, −*z*+1.
