# Supplementary figures and images for: Crystal structure of (E)-3-allyl-2-sulfanyl­idene-5-[(thio­phen-2-yl)methyl­idene]thia­zolidin-4-one
Source: Acta Crystallogr E Crystallogr Commun. 2015 May 30;71(Pt 6):o433–4. doi: 10.1107/S2056989015010166 (PMC4459307; doi:10.1107/S2056989015010166)

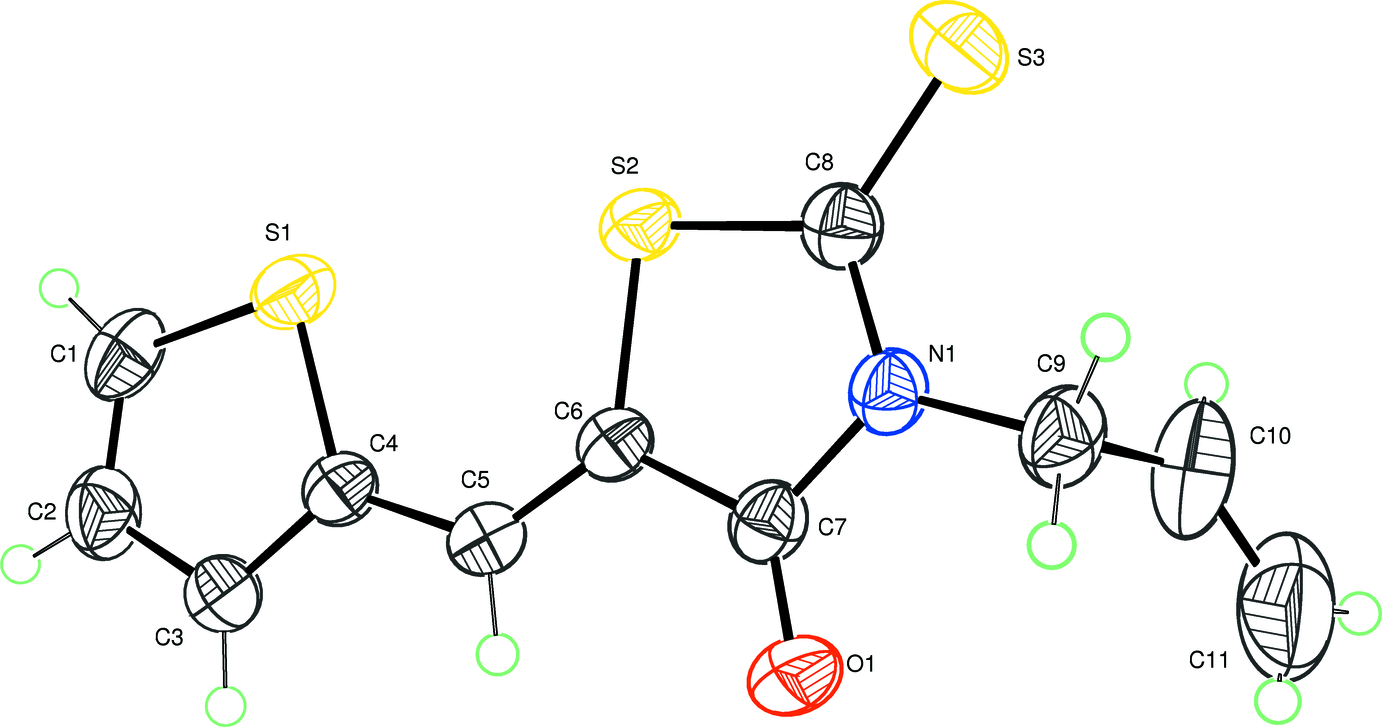

Supplement: Supplementary file 4 [file e-71-0o433-fig1.tif]

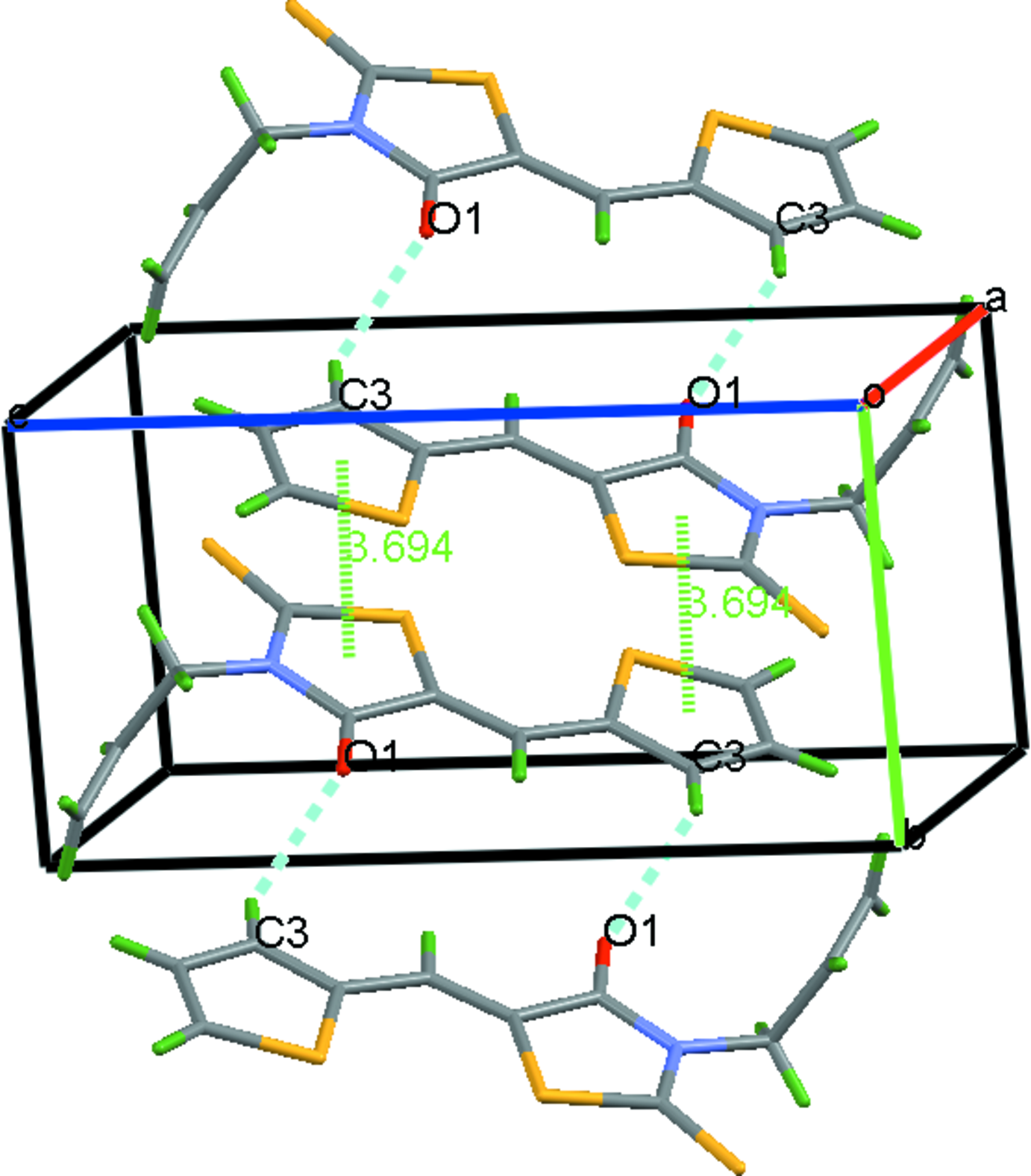

Supplement: Supplementary file 5 [file e-71-0o433-fig2.tif]
